# VEGF stimulated the angiogenesis by promoting the mitochondrial functions

**DOI:** 10.18632/oncotarget.20331

**Published:** 2017-08-18

**Authors:** Dongqing Guo, Qiyan Wang, Chun Li, Yong Wang, Xing Chen

**Affiliations:** ^1^ School of Life Sciences, Beijing University of Chinese Medicine, Beijing, 100029, China; ^2^ Modern Research Center for Traditional Chinese Medicine, Beijing University of Chinese Medicine, Beijing, 100029, China; ^3^ School of Information and Control Engineering, China University of Mining and Technology, Xuzhou, 221116, China

**Keywords:** VEGF, angiogenesis, mitochondria

## Abstract

The vascular endothelial growth factor (VEGF) signaling pathway involved in angiogenesis which plays a pivotal role in normal development and also represents a major therapeutic target for tumors and intraocular neovascular disorders. The aims of the present study were to evaluate the effects of VEGF on endothelial cells and clarify the mechanism. Here, we showed that VEGF significantly stimulated the proliferation, migration and cell cycle of endothelial cells, and it also induced tube formation *in vitro* significantly. What's more, the mitochondrial functions were enhanced in response to VEGF, including mitochondrial oxidative respiration and intracellular ATP levels. The reactive oxygen species (ROS) production decreased, while the enzymes of ROS defence system, including catalase and glutathione peroxidase (GPX1), whose expression both increased in the VEGF stimulation. VEGF activated mammalian target of rapamycinm (mTOR) signaling pathway to promote the function of mitochondria. Rapamycin, the inhibitor of mTOR pathway could inhibit the proliferation and cell cycle induced by VEGF. In summary, our study identified that VEGF promoted the angiogenesis and provided evidence for mitochondria as new therapeutic target of VEGF signaling in the angiogenic vascular disorders.

## INTRODUCTION

Angiogenesis is the formation of new blood vessels that sprouting from the pre-existing primitive vessels and occurs during normal development, reproduction and tissue repairing [1]. However, excessive angiogenesis is related with some disease states, such as cancer [2], diabetic retinopathy [3], preeclampsia [4] and so on. The proliferation and migration of endothelial cells into the extracellular matrix towards the angiogenic factors play central roles in the process of angiogenesis. Targeting ECs to prevent dysfunction or inhibit pathological angiogenesis is potentially beneficial for a wide variety of diseases, and the current studies focus primarily on growth factors, receptors and signaling molecules. Of the growth factors involved in the angiogenesis, vascular endothelial growth factor (VEGF) is a key regulator. VEGF family currently includes six known members: VEGF-A through E and placental growth factor. VEGF-A is most prevalent and consists of five isoforms of which VEGF165 is the predominant molecular species [5]. VEGF induced by hypoxia [6], initiates the process of angiogenesis, via the activation of two receptor tyrosine kinases (RTKs), VEGF receptor-1 (VEGFR1) and VEGF receptor-2 (VEGFR-2)[7]. There are three core signaling pathways on the downstream of VEGFR2. They are the PI3K (phosphatidylinositol 3-kinase)–Akt (Protein Kinase B) pathway, which promotes cell survival and vascular permeability, the Raf-MEK (mitogen-activated or extracellular signal-regulated protein kinase kinase)-MAPK (mitogen-activated protein kinase) pathway, which activates cell proliferation, and the Src-FAK (focal adhesion kinase) pathway, which increases cell motility [8]. MicroRNAs also involved in the VEGF pathways. MiR-26a serveds as a critical regulator of VEGF-mediated angiogenesis through directly targeting Nogo-B receptor (NgBR) in endothelial cells [9]. MiR-29a lowered VEGF production and angiogenic activities in synovial fibroblasts through targeting the 3'-UTR of VEGF, thus miR-29a deficiency exacerbated synovitis pathogenesis in the end-stage OA knees [10]. Pleiotropic action of miR-21 induced the expression of pERK, HIF-1α, and VEGF in the high glucose condition by simultaneously targeting SPRY1, SMAD7, and PTEN in ARPE-19 cells [11].

Targeting endothelial cell metabolism to treat angiogenesis and endothelial cell dysfunction is becoming a novel therapeutic strategy. Despite their close proximity to oxygenated blood, ECs rely on glycolysis instead of oxidative metabolism for adenosine triphosphate (ATP) production [12]. The loss of the glycolytic enzyme PFKFB3 (fructose-2, 6-biphosphatase 3) in ECs impaired vessel formation [13]. In contrast to cardiac myocytes and other cell types, energy requirements in the endothelial cells are relatively low, so mitochondria in ECs play a vital role in signaling cellular responses to environmental cues [14], including biogenesis, dynamics, mitophagy, ROS production, calcium homeostasis, regulated cell death, and heme synthesis. But the effects of VEGF on mitochondria are still unknown.

To better understand the VEGF pathways will help us evaluate the mechanism of angiogenesis and provide new targets for therapy. In this present paper, we evaluated the effects of VEGF on the proliferation, migration, cell cycle and angiogenesis of endothelial cells *in vitro*. We also explored the mechanism of VEGF based on the mitochondria and expected to find the new targets for angiogenic vascular disorders.

## RESULTS

### VEGF significantly promoted the proliferation, migration, angiogenesis and cell cycle of endothelial cells

**Figure 1:**
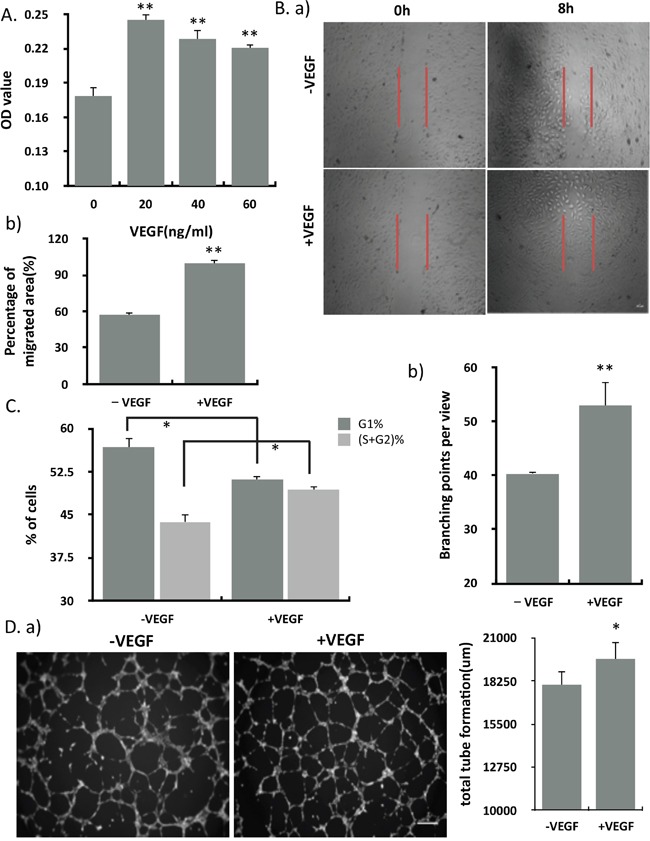
VEGF significantly promoted the proliferation, migration, angiogenesis and cell cycle of endothelial cells **(A)** The proliferation of endothelial cells treated with different concentration of VEGF (n=12). **(B)** Migration of HUVEC in the absence and presence of VEGF (20 ng/ml) was assessed by wound healing assay. a) Representative micrographs of wound healing assays at 0 hrs and 8 hrs after creating a wound field. Scale bar, 200um. b) Quantitative assessment of percentage of cells migrating into the wound field (n=3). **(C)** PI staining followed by flow cytometry was used to assess cell cycle. Cell cycle phase length was calculated from the percentage of cells present in each cell cycle phase (n=3). **(D)** ECs were seeded on extracellular matrigel and exposed to VEGF treatments. a) Representative bright field micrographs of EC matrigel angiogenesis after 5h of VEGF treatment. b) Quantitative analysis of the number of branching points and total tube length (n=3). Scale bar, 100um. Results represented mean ± SEM of n independent experiments. **P<0.01, *P<0.05, 1-way ANOVA.

To investigate the effects of VEGF on endothelial cells, HUVECs were treated with VEGF at different concentrations *in vitro*. As measured by MTT assay, VEGF significantly promoted the proliferation of HUVECs at the concentration of 20 nM (Figure 1A). HUVECs also showed significant increased migration in response to 20 nM VEGF. The increased migration rate is nearly two fold of the control (Figure 1B). As expected, ECs showed a reduced of cells in G1-phase and an accumulation of cells in S and G2 phase, correlating with the increased proliferation in response to VEGF (Figure 1C). We used a matrigel angiogenesis assay to explore the effects of VEGF on the *in vitro* tube formation and found that VEGF alone promoted the ability of HUVECs to form tube-like structures *in vitro* (Figure 1D).

### Mitochondrial function was enhanced in response to VEGF

**Figure 2:**
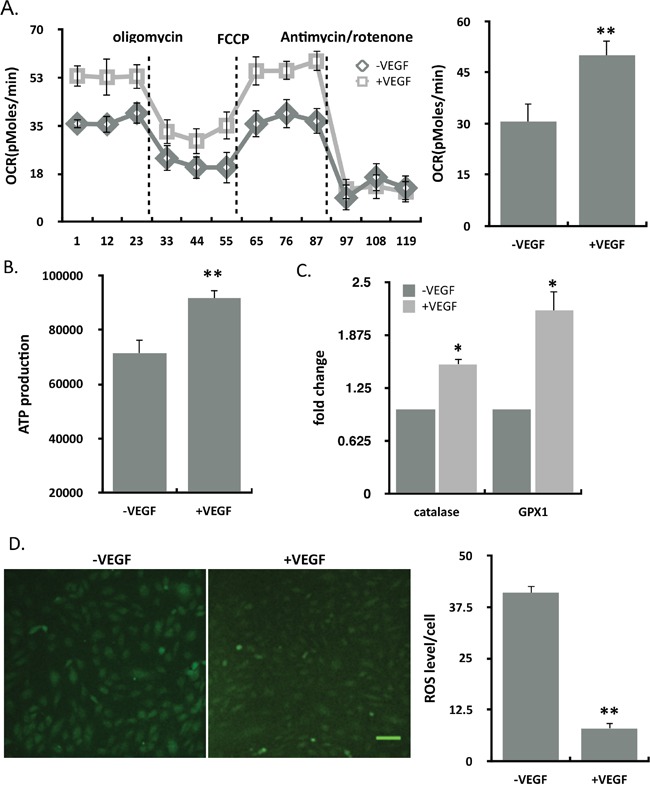
VEGF enhanced the mitochondrial function **(A)** Oxygen consumption rate (OCR) in control and VEGF-treated ECs using by seahorse. Statistics of FCCP-coupled OCR was shown in the graph (n=10). **(B)** Intracellular ATP levels in control and VEGF-treated ECs (n=16). **(C)** Catalase and GPX1 mRNA were analysed by means of quantitative RT-PCR. Relative expression values of the VEGF-untreated cells were taken as 1.0 (n=4). **(D)** ROS staining was performed using DCFH-DA in ECs in the absence and presence of VEGF. Left: Representative images of ROS staining; right: Statistics of ROS fluorescence intensity in per cell (n=8). Scale bar, 100um. Results represented mean ± SEM of n independent experiments. **P<0.01, *P<0.05, 1-way ANOVA.

ECs relay on glycolysis rather than on oxidative phosphorylation in the vessel sprouting. To explore which mechanism VEGF promotes ECs, we found that mitochondrial oxidative respiration was enhanced in ECs treated with VEGF (Figure 2A). Intracellular ATP levels increased (Figure 2B) and the ROS production decreased (Figure 2D) in VEGF-treated ECs. Moreover, the genes of ROS defence system, including catalase and glutathione peroxidase (GPX1), whose expression both increased in the VEGF stimulation (Figure 2C). Overall, VEGF protected endothelial cells by enhancing mitochondrial function.

### VEGF activated mTOR signaling pathway

**Figure 3:**
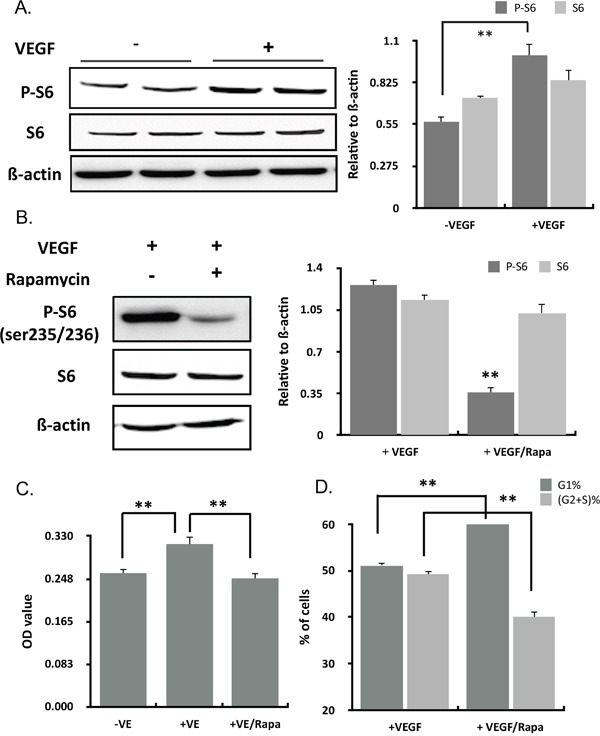
VEGF activated mTOR signaling pathway **(A)** HUVECs cultured in M199 medium with 5%FBS were induced with VEGF for 24 hours. P-S6 and S6 were determined by western blots. **(B)** HUVECs cultured in M199 medium with 5%FBS were induced with VEGF and rapamycin for 24 hours. P-S6 and S6 were determined by western blots. **(C)** Cell proliferation in cultured HUVECs treated with VEGF and the inhibitor of mTOR signaling, rapamycin (50 nM) for 24 hours (n=12). **(D)** Cell cycle in cultured HUVECs treated with VEGF and an inhibitor of mTOR signaling, rapamycin (50 nM) for 24h determining by PI staining (n=3).

mTOR is a central regulator of cellular growth and inhibition of mTOR by Rapamycin decreased oxygen consumption and mitochondrial capacity. To determine the molecular basis for VEGF effects on the mitochondria of ECs, we investigated the role of mTOR signaling in the VEGF stimulation. VEGF could activate the phosphorylation of the ribosomal S6 protein in ECs, a best-characterised mTOR substrate, while expression of total S6 had no change (Figure 3A). Rapamycin, the mTOR inhibitor, could inhibit the increasing of P-S6 (Figure 3B) and the proliferation induced by VEGF (Figure 3C), accompanying with an accumulation of cells in G1 and reduced S+G2 phase (Figure 3D). It was concluded that VEGF activated mTOR signaling pathway.

## DISCUSSION

In the present study, we found that VEGF significant stimulated endothelial cells proliferation, migration, cell cycle and tube formation *in vitro*. We further investigated the mechanism and found that mitochondrial functions were enhanced induced by VEGF. Mitochondria in endothelial cells primarily function in signaling cellular responses to environmental cues not to produce ATP [14]. Mitochondria dysfunction was induced by many diseases in the endothelium, thus interventions that restore mitochondria function might be protective [15].

Our results showed that mitochondrial oxidative respiration and intracellular ATP levels increased in response to VEGF. The ROS production decreased, while the genes of ROS defence system, including catalase and glutathione peroxidase (GPX1), whose expression both increased in the VEGF stimulation. VEGF activated mTOR signaling pathway to promote the mitochondria functions which may become a potential regulator of mitochondria function. Rapamycin, the inhibitor of mTOR pathway could inhibit the proliferation and cell cycle induced by VEGF. mTOR signaling pathway regulates cell metabolism and energy homeostasis [16] and positively regulates PGC-1α expression [17], a coactivator that controls mitochondrial biogenesis. PGC-1α could enhance oxidative phosphorylation, mitochondrial biogenesis and the oxygen consumption rate in cancer cells [18]. ROS accumulation in cancer cells induces expression of PGC-1å/ß to promote detoxification through direct induction of superoxide dismutase 2 (SOD2), catalase and glutathione peroxidase [19].

It has been reported that one class of anti-angiogenic agents used in treating cancer directly inhibit endothelial cells function and induce endothelial cells death by targeting mitochondria. 10-(6’-plastoquinonyl) decyltriphenylphosphonium (SkQ1) is a new antioxidant with the highest mitochondria membrane penetrating ability and it could modulate the tumor angiogenesis [20]. Vitamin E analogues are first recognized for their selective toxicity to cancer cells. Vitamin E analogues a-TOS and a-TEA bind to Complex II in the inner-mitochondrial membrane, leading to displacement of ubiquinone and the generation of reactive oxygen species [21]. Paclitaxel (Taxol) is potent chemotherapeutic used in the treatment of ovarian, lung and breast malignancies. It could directly interact with isolated mitochondria, initiating the MPTP and inducing cytochrome C release [22]. Thus mitochondria are the focal point for a variety of angiogenic signals [23].

In this paper, *in vivo* experiments were lacking. To develop robust combinatory cancer hallmark–based gene signature sets (CSS sets) that more accurately predict prognosis [24], so we believe that it would be a good idea to take the RNA-seq or microarray to identify if the expression of the VEGF and the related mitochondrial genes could predict cancer recurrence. What's more, the effects of other growth factors, such as VEGF-E and TGF-ß need to be explored in the further.

In summary, our findings provide the evidence that mitochondria play a dominant role in the stimulation of VEGF. The mitochondrial functions were enhanced. This is the first time to demonstrate the mechanism of VEGF signaling pathway based on the mitochondria. It is of significant importance and may provide new mechanistic insights into angiogenic vascular disorders.

## MATERIALS AND METHODS

### Cell culture and chemicals

Human umbilical vein endothelial cells (HUVECs) were purchased from PromoCell (Germany). HUVECs were grown in endothelial cells medium (Promocell) and maintained at 37°C and 5% CO_2_. Cells were used within five passages. The information of drugs were shown in the below table.

**Table:** 

Name	Corporation	SKU
DCFH-DA	Sigma	D6883
rapamycin	Sigma	V900930
MTT	Sigma	M2128
matrigel	BD	356230
CellTiter-Glo® Luminescent Cell Viability Assay	Promega	G7570
anti-Phospho-S6 Ribosomal Protein	Cell Signaling	#2211
anti-S6 Ribosomal Protein (5G10)	Cell Signaling	#2217
anti-ß-actin	Beijing TDY Biotech LTD	#M009
HRP-conjugated secondary mouse antibody	Beijing TDY Biotech LTD	#E009
HRP-conjugated secondary rabbit antibody	Beijing TDY Biotech LTD	#E011
endothelial cells medium	PromoCell	C-22010
TransStart Green qPCR Supermix	Transgene	AQ101-02
HUVEC	PromoCell	C-12206

### Cell proliferation assay

HUVECs were seeded into 96-well cell culture plates and allowed them to adhere for 12h. After incubation with various concentration of VEGF for 24h, 10ul of MTT was added to each well for 3h incubation. Subsequently, cells were dissolved by 150 mL of DMSO, mixed and measured the absorbance (A) by Multiskan (Thermo) at 562 nm.

### Scratch wound assay

HUVECs were deprived of serum for 12h and scratched with a sterile P200 pipette tip according to a paradigm previously described [25]. After removal of the debris by repeated washes, cells were subjected to VEGF treatment and scratch wound closure was monitored by phase microscopy capturing images of the same field with a 4X objective at 0h and 8h. The percentage of migrated area was calculated with “Image J” software.

### PI staining for cell cycle

HUVECs were pretreated with VEGF for 24h. Cells were trypsinized and fixed by -80°C 70% ethanol for 24 hours. And then cells were centrifuged and stained with PI buffer in dark at 37°C for 30min. Cell cycle was evaluated by flow cytometry according to the manufacturer's protocol (BD PharMingen, San Diego, CA, USA). PI buffer(200μL) including: 160μL 0.1% Triton X-100/PBS, 20μL 0.5 mg/ml PI, 20μL 10 mg/ml RNase A.

### *In vitro* angiogenesis assay

Angiogenic potential was assessed by the spontaneous formation of capillary-like structures by HUVECs on growth factor–reduced Matrigel. HUVECs (1×10^4^ cells/ well) were seeded in 96-well matrigel-coated plates using completed medium. After 1 hours, cells were stimulated with 20 nM VEGF. And after 4-5 hours, cells were observed with a Nikon inverted microscope and images recorded and analysed by using Image Pro-Plus image analysis software (Media Cybernetics).

### Measurement of oxygen consumption rate

Cellular OXPHOS was monitored using the Seahorse Bioscience Extracellular Flux Analyzer (XF24, Seahorse Bioscience Inc., North Billerica, MA, USA) by measuring the OCR (indicative of respiration). Briefly, 3000-5000 cells were seeded in 24-well plates designed for XF24 in 150μL of appropriate growth media and incubated overnight, then with different treatments. Before measurements, cells were washed with unbuffered media once, then immersed in 675μL unbuffered media and incubated in the absence of CO_2_ for 1h at 37°C. OCR was then measured in a typical 8-min cycle of mix (2-4 min), dwell (2 min) and measure (2-4 min) as recommended by Seahorse Bioscience.

### Intracellular ATP content measurements

CellTiter-Glo Luminescent Cell Viability Assay was used according to the manufacturer's instructions. Luminescence signal was measured using synergy HT fluorometer (Bio Tek, Winooski, VT, USA). The values were normalized to control for each corresponding point.

### Measurements of intracellular reactive oxygen species

Intracellular ROS was quantified by a laser confocal microscope using the fluorescent probe, 2’, 7’-dichlorodihydrofluorescein diacetate (DCFH-DA). HUVECs were incubated in phenol red-free media supplemented with 10μM DCFH-DA in dark for 20 min at 37°C. They were then washed twice with PBS and immediately observed.

### Quantitative reverse transcription PCR

Quantitative reverse transcription PCR (qRT-PCR) was carried out using the following oligonucleotides and PCR conditions: 2 minutes at 95°C (20 seconds at 95°C, 20 seconds at the annealing temp, and 30 seconds at 72°C) × 35, and 10 minutes at 72°C. Primers were as follows: ß-actin, forward: 5′-CATCACTATTGGCAACGAG-C-3′, reverse: 5′-ACGCAGCTCAGTAACAGTCC-3′; catalase, forward: 5′-TGGAGCTGGTAACCCAGTAGG-3′, reverse: 5′-CCTTTGCCTTGGAGTATTTGGTA-3′;GPX1, forward: 5′-CAGTCGGTGTATGCCTTCTCG-3′, reverse: 5′-GAGGGACGCCACATTCTCG-3′.

### Western blots analysis

Cell lysate proteins were separated by SDS-PAGE (10% gels) and transferred to nitrocellulose membranes. Membranes were blocked in Tris-buffered saline with 0.05% Tween 20 (TBST) containing 5% nonfat dry milk powder for 1 hour. Western blots were probed with primary antibodies for 1 hour, washed 3 times with TBST, and then incubated with the appropriate secondary antibodies for 30 minutes. Membranes were then washed with TBST 3 times, before developing with SuperSignal West Dura chemiluminescent substrate. The following antibodies were used: rabbit anti-Phospho-S6 Ribosomal Protein (Ser235/236) (Cell Signaling, #2211, 1:1000), rabbit anti-S6 Ribosomal Protein (5G10) (Cell Signaling, #2217, 1:1000), mouse anti-ß-actin (Beijing TDY Biotech LTD, #M009, 1:5000), HRP-conjugated secondary mouse (Beijing TDY Biotech LTD, #E009, 1:5000) and rabbit antibodies (Beijing TDY Biotech LTD, #E011, 1:5000).

### Statistical analyses

All data were derived from ≥3 independent experiments. Statistical analyses were conducted using SPSS version 19.0 (IBM SPSS, Armonk, NY, USA). Values were calculated as mean ± standard error of the mean. Significant differences between the groups were determined using one-way analysis of variance test. A p-value P<0.05 was considered to indicate a statistically significant difference.

## Abbreviations

VEGF: vascular endothelial growth factor
ECs: endothelial cells
HUVECs: Human umbilical vein endothelial cells
ROS: reactive oxygen species
GPX1: glutathione peroxidase (GPX1)
mTOR: mammalian target of rapamycinm
ATP: adenosine triphosphate
PFKFB3: fructose-2, 6-biphosphatase 3
